# Spatiotemporal analysis of drug-resistant TB patients registered in selected districts of Karnataka, South India: a cross-sectional study

**DOI:** 10.1186/s41182-020-00199-7

**Published:** 2020-03-10

**Authors:** Basavaraj Poojar, K. Ashok Shenoy, Poonam R. Naik, Ashwin Kamath, Jaya Prasad Tripathy, P. Prasanna Mithra, Mukta N. Chowta, M. N. Badarudeen, Narasimhaswamy Nagalakshmi, Vivek Sharma, Amrita N. Shamanewadi, Pruthu Thekkur

**Affiliations:** 1 Department of Pharmacology, Kasturba Medical College, Mangalore, Manipal Academy of Higher Education (MAHE), Manipal, Karnataka India; 2 Department of Community Medicine, Yenepoya Medical College, Mangalore, Yenepoya (Deemed to be University), Mangalore, Karnataka India; 3 Centre for Operational Research, International Union Against Tuberculosis and Lung Disease, Paris, France; 4 Department of Community Medicine, Kasturba Medical College, Mangalore, Manipal Academy of Higher Education (MAHE), Manipal, Karnataka India; 5 District TB Center, Mangalore, India; 6 Department of Microbiology, Melaka Manipal Medical College, Manipal Academy of Higher Education, Manipal, India; 7 Department of Microbiology and Immunology, College of Medicine, American University of Antigua, St John’s, Antigua and Barbuda; 8 Tuberculosis Health Action and Learning Initiative (THALI), JSI India, New Delhi, West Bengal India; 9 Department of Community Medicine, MVJ Medical College and Research Hospital (MVJ&MCRH) Hoskote, Bangalore, India

**Keywords:** Spatial heterogeneity, Drug-resistant tuberculosis, GIS, Spatial analysis, Geographic mapping, Spatial epidemiology

## Abstract

**Background:**

Tuberculosis (TB) depicts heterogeneous spatial patterns with geographical aggregation of TB cases due to either ongoing person-to-person transmission or reactivation of latent infection in a community sharing risk factor. In this regard, we aimed to assess the spatiotemporal aggregation of drug-resistant TB (DR-TB) patients notified to the national TB program (NTP) from 2015 to 2018 in selected districts of Karnataka, South India.

**Methods:**

This was a cross-sectional study among DR-TB patients notified from Dakshina Kannada, Udupi, and Chikamagalur districts of the state of Karnataka. Clinico-demographic details were extracted from treatment cards. The registered addresses of the patients were geocoded (latitude and longitude) using Google Earth. Using the QGIS software, spot map, heat maps and grid maps 25 km^2^ with more than the expected count of DR-TB patients were constructed.

**Results:**

Of the total 507 patients studied, 376 (74%) were males and the mean (standard deviation) age of the study participants was 41.4 (13.9) years. From 2015 to 2018, the number of patients increased from 85 to 209 per year, the area of aggregation in square kilometers increased from 113.6 to 205.7, and the number of rectangular grids with more than the expected DR-TB patients (> 1) increased from 12 to 47.

**Conclusions:**

The increase in the number of DR-TB patients, area of aggregation, and grids with more than the expected count is a cause for concern. The NTP can use routine programmatic data to develop maps to identify areas of aggregation of disease for targeted TB control activities.

## Introduction

Globally, tuberculosis (TB) remains a major public health problem of concern with an estimated 10 million incident TB patients and 1.3 million deaths due to TB in the year 2017. Multidrug-resistant tuberculosis (MDR-TB, TB resistant to isoniazid and rifampicin) and extensively drug-resistant tuberculosis (XDR-TB, MDR-TB plus resistance to any fluoroquinolones and at least one second-line injectable drug) have emerged globally and pose a threat to TB control efforts. The World Health Organization (WHO) estimated about 330,000 incident multidrug-resistant TB or rifampicin-resistant TB (MDR/RR-TB) patients globally in the year 2017 [1]. However, only 54% of the estimated were notified to the national TB program (NTP). Thus, almost half of the estimated MDR/RR-TB patients went either undiagnosed or diagnosed but not reported [2].

India is one of the 30 high-burden TB countries and has a triple burden of TB, TB/HIV, and MDR-TB. In 2017, it was estimated that there were 65,000 MDR/RR-TB patients in the country with a prevalence of 2.8% among new TB cases and 12% among previously treated TB cases. Similar to global trends, there is a substantial gap in the detection and treatment of MDR/RR-TB patients in India. Of the estimated, only about 40% of the MDR/RR-TB patients are identified and initiated on treatment [2]. As the majority of patients are missed, identifying geographical areas with a high incidence of disease and adopting active case finding in such areas could help to reduce detection gap among DR-TB patients.

TB depicts heterogeneous spatial patterns with localized aggregation of cases due to either ongoing person-to-person transmission [3] or reactivation of latent infection in a community sharing risk factor. Biological (malnutrition, HIV infection, and age distribution) and social determinants (poverty and behavioral risk factors) of TB are aggregated in geographical areas and eventually can lead to aggregation of TB patients in such areas. The previous studies on geospatial patterns of MDR-TB in Peru [4], Moldova [5], and Georgia [6] have shown MDR-TB patients to be aggregated within geographical areas. If the DR-TB is characterized by areas of concentrated risk rather than spatially uniform risk, intensitfied control activities at areas with concentrated risk may be efficient than adopting blanket approach for control of the disease.

Though geospatial analysis, identifying areas with the aggregation of TB patients, and focused TB control activities in such areas may be theoretically beneficial, such explorations are scarce. As a first step, there is a need for assessing the geospatial distribution of drug-resistant TB locally to detect whether there is any geographical aggregation of DR-TB patients using routine program data. However, the inherent difficulties in geotagging the location of the patient and technical challenges in handling such data have limited the use of geospatial epidemiology in TB control efforts. Hence, as a desk review, we explored the possibility of geocoding the address of DR-TB patients using Google Maps and constructing the spatial heterogeneity maps. We geocoded all DR-TB patients treated under the Revised National Tuberculosis Control Program (RNTCP) in three selected districts of Karnataka from 2015 to 2018 to depict the spatiotemporal pattern of these patients.

## Methodology

### Study design

It is a cross-sectional descriptive study using secondary data collected routinely by the RNTCP of India.

### Study setting

#### General setting

Karnataka is a southern state of India. The approximate population of Karnataka is 66.8 million with a sex ratio of 973 females to 1000 males [7]. The RNTCP was universalized in the state by 2002. In 2016, the state of Karnataka had an annual TB notification rate of 123/100,000 individuals. Of the total cases notified, 79% (47,145) are new TB patients, and 29% (12,587) are previously treated. During the same year, the state had notified 1099, of which, 925 initiated DR-TB treatment [8].

#### Specific setting

##### Study site

**Fig. 1:**
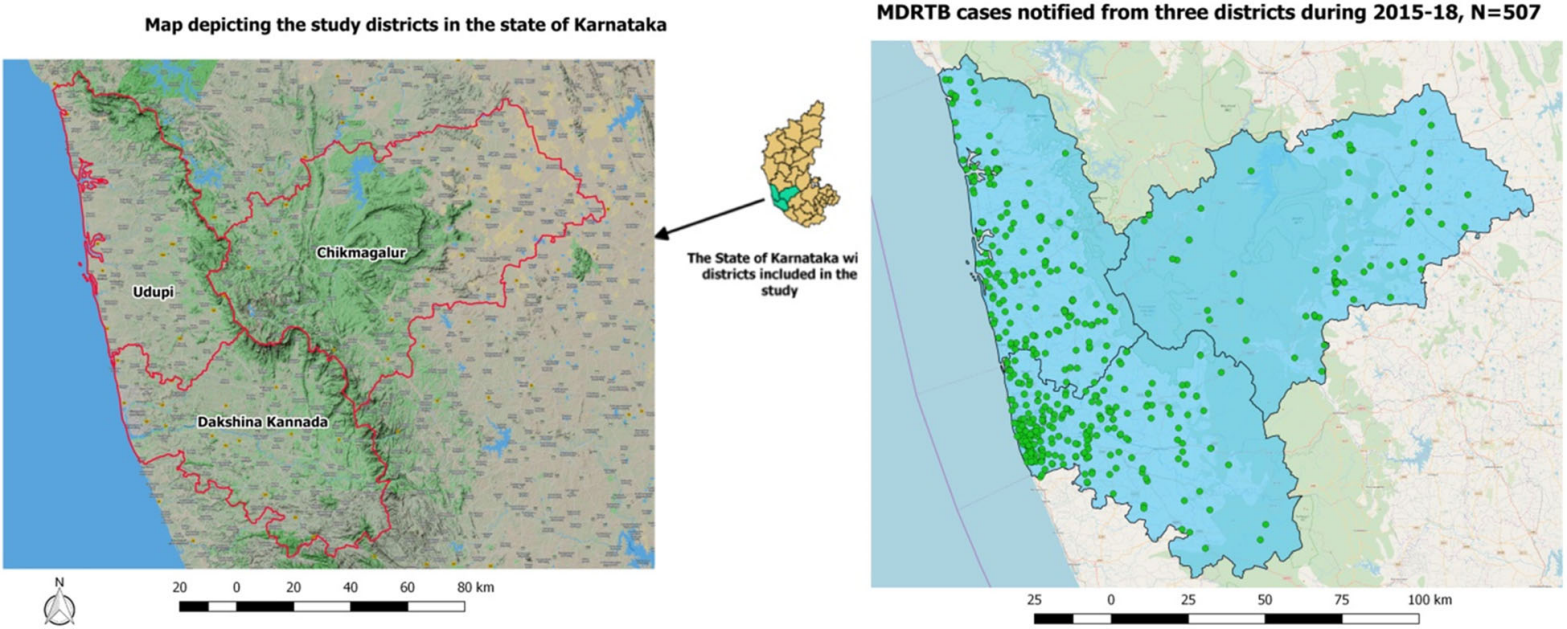
The map depicting the study districts and spot map of drug-resistant TB patients in the state of Karnataka, India

The study was conducted in the DR-TB center of Mangalore, Karnataka. The DR-TB center caters to all the DR-TB patients diagnosed in three districts, namely Dakshina Kannada, Chikmagalur, and Udupi (Fig. 1). According to the 2011 census [9], Dakshina Kannada district had a population of 2 million [10] and Chikmagalur and Udupi districts had a population of 1.1 million each [11, 12]. The Revised National Tuberculosis Control Program (RNTCP) has 21 tuberculosis units (TUs) and 74 designated microscopy centers (DMCs) providing TB diagnosis and treatment support.

### Diagnosis of DR-TB

The Programmatic Management of Drug-Resistant TB (PMDT) guideline was followed for the diagnosis and treatment of DR-TB in the study site. From 2015 to 2017, the criteria-C was adopted for identifying presumptive MDR-TB patients [13]. All the presumptive MDR-TB patients were offered both phenotypic (culture and drug sensitivity) and molecular (Xpert MTB/RIF assay) drug susceptibility testing (DST) for detecting drug resistance to first-line drugs. From January 2018, universal drug susceptibility testing (UDST) was implemented in the study setting [8]. As per UDST, all the patients diagnosed with TB were provided upfront DST using Xpert MTB/Rif assay [14].

#### DR-TB treatment initiation, follow-up, and treatment outcomes

All the diagnosed DR-TB patients from the districts included in the study were referred to the DR-TB center at Mangalore for pretreatment evaluation and initiation of treatment. The patients reaching the DR-TB center were registered for care with a unique DR-TB registration number. The socio-demographic and clinical details, along with the address and phone number of the patient, were recorded in the DR-TB register by the staff nurse. Also, the PMDT treatment card was issued for each patient and had the same details recorded in it.

At the DR-TB center, the patients were hospitalized for pretreatment evaluation and treatment initiation. All DR-TB patients were prescribed drugs based on PMDT guidelines. After initiating the therapy, patients were monitored at the DR-TB center for 1 to 2 weeks. Once a patient is stable and tolerating the second-line drugs, then he/she was referred to the peripheral health institute (PHI) nearest to patient residence for further care and monitoring with ambulatory DOTS-Plus treatment at PHI. All the details of the treatment course in the DR-TB center and referral details were updated in the DR-TB treatment card and DR-TB register.

#### Diagnosis of DR-TB and treatment by private providers

The DR-TB patients diagnosed by private providers are notified to RNTCP. As the treatment of DR-TB is complicated, majority of the patients are referred to public health facilities for treatment and eventually initiated treatment at the DR-TB center. However, a few patients might continue to be treated by private providers.

### Study population

All DR-TB patients initiated on TB treatment from a DR-TB center at Mangalore from January 2015 to December 2018 were included.

### Data variables, sources of data, and data collection

Data on socio-demographic and clinical characteristics and drug-resistant pattern like PMDT number, age, gender (male/female/others), village/ward, taluk, district, name of TU, name of PHI, DOTS provider, date of DST, date of DST results, date of registration, type of TB (new/treatment after loss to follow-up/treatment after failure/recurrent/relapse), site of TB (pulmonary/extra-pulmonary), type of DST used for diagnosis (CDST/Xpert MTB/RIF assay), molecular test results (not done/Mtb+/Rif+/Mtb+/Rif−/Mtb−/MTB+ Rif indeterminate), type of DR-TB (RR-TB/MDR-TB/XDR-TB), resistance to isoniazid (yes/no/not available), resistance to ethambutol (yes/no/not available), resistance to pyrazinamide (yes/no/not available), resistance to streptomycin (yes/no/not available), resistance to fluoroquinolones (yes/no/not available), resistance to kanamycin (yes/no/not available), HIV status (yes/no/not available), ART status (yes/no/not available/not applicable), weight at initiation of treatment, tobacco use (yes/no/not available), and alcohol use (yes/no/not available) were extracted from DR-TB treatment card and DR-TB register maintained at DR-TB center.

#### Geocoding

The geocode (latitude and longitude) of each patient was obtained based on the registered address using Google Earth pro version 7.3.2.5776 application. The village/ward/street of the patient was used to geocode the address [15–17]. The details like taluk, district, and PHI were used to locate the village/ward/street on the geospatial map. In case of non-availability of the village/ward/street, the support was sought from Senior Treatment Supervisor (STS) of the TU or PHI to which the patient was referred for DOTS-Plus. If the village/ward/street provided in the address is not found on Google Maps, the nearest village as suggested by the STS or staff of PHI was geocoded. The geocodes were noted in decimal degrees format with a minimum of five digits after the decimal point [18].

### Data entry and analysis

Data were double entered and validated using EpiData Entry software (EpiData Association, Odense, Denmark) and analyzed using EpiData analysis (version 2.2.2.182, EpiData Association, Odense, Denmark) and Stata version 12.0 (STATA Corp., College, TX, USA). Socio-demographic and clinical characteristics and drug-resistant patterns were summarized using numbers and percentages.

The QGIS version 2.18.15 (QGIS Developer team, Las Palmas (2016)) was used to plot the drug-resistant TB patients, and a map of geospatial distribution was constructed. The heat maps depicting spatial heterogeneity [19] were constructed for the year 2015 to 2018. The quadratic weight was used as a density measure [20], and the heat maps [5, 20] were constructed with a buffer radius appropriate to the layer units [21]. The heat map constructed depicts the geographical aggregation of the DR-TB patients but not the relatedness of the cases.

The grid maps were constructed with each grid of 25 km^2^. Within each grid, the number of DR-TB cases was counted. Those cases with more than the expected number of DR-TB cases were colored red. The expected DR-TB patients per grid were calculated with a population density of 282 per square kilometer in three districts, and the estimated number of TB patients per 25 km^2^ is 15 (considering estimated annual TB incidence of 210/1,000,000 in the country) [14]. The expected number of DR-TB is 1 per 25 km^2^ assuming the proportion of DR-TB cases among TB patients to be 6%.

## Results

**Fig. 2:**
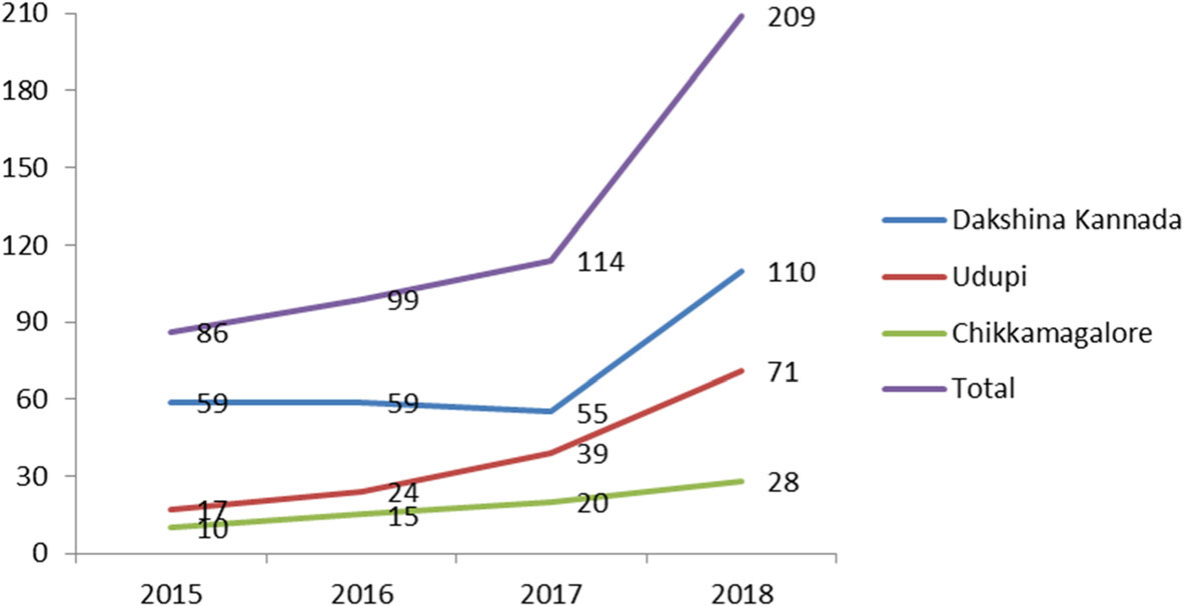
The trends in DR-TB patients notified from the selected districts of Karnataka during 2015 to 2018, *N* = 507

In total, 507 DR-TB patients were notified and initiated on treatment from 2015 to 2018 in the study districts. The mean (SD) age of the study participants was 41.4 (13.9), and 376 (74%) were males. Of the total, about 452 (89%) of the DR-TB patients were new TB patients. The DR-TB patient characteristics included in the study are shown in Table 1. During the study period, the number of DR-TB cases increased from 85 in the year 2015 to 209 in 2018. Figure 2 depicts the trends in notified DR-TB patients across the study districts.

**Table 1:** Socio-demographic and clinical characteristics of DR-TB patients initiated on treatment under the national TB program in three selected districts of Karnataka during 2015 to 2018, *N* = 507

Characteristics	Frequency	Percentage^*^
Age (in years)		
0–14	2	0.3
15–24	51	10.0
25–34	123	24.2
35–44	116	22.8
45–54	110	21.6
55–64	64	12.6
≥ 65	36	7.1
Not recorded	5	0.9
Gender		
Male	377	74.3
Female	129	25.4
Not recorded	1	0.1
District		
Dakshina Kannada	283	55.8
Udupi	151	29.7
Chikamagalur	73	14.3
Year (total number of cases)		
2015	85	16.7
2016	99	19.5
2017	114	22.4
2018	209	41.2
Type of TB		
New	340	67.0
Retreatment after loss to follow-up	43	8.4
Retreatment after failure	55	10.8
Retreatment/relapse	69	13.6
DST used for diagnosis		
CDST	30	5.9
Xpert MTB	260	51.2
LPA	216	42.6
Not recorded	1	0.1
HIV status		
Positive	18	3.5
Negative	346	68.2
Unknown	4	0.7
Not recorded	139	27.4
ART status (*N* = 18)		
On ART	17	94.4
Not on ART	1	5.6

*Abbreviation*: *DR-TB* drug-resistant tuberculosis, *DST* drug sensitivity testing, *CDST* culture and drug susceptibility test, *LPA* line probe assay, *MTB Mycobacterium tuberculosis*, *Rif* rifampicin, *HIV* human immunodeficiency virus

*Column percentage

**Fig. 3:**
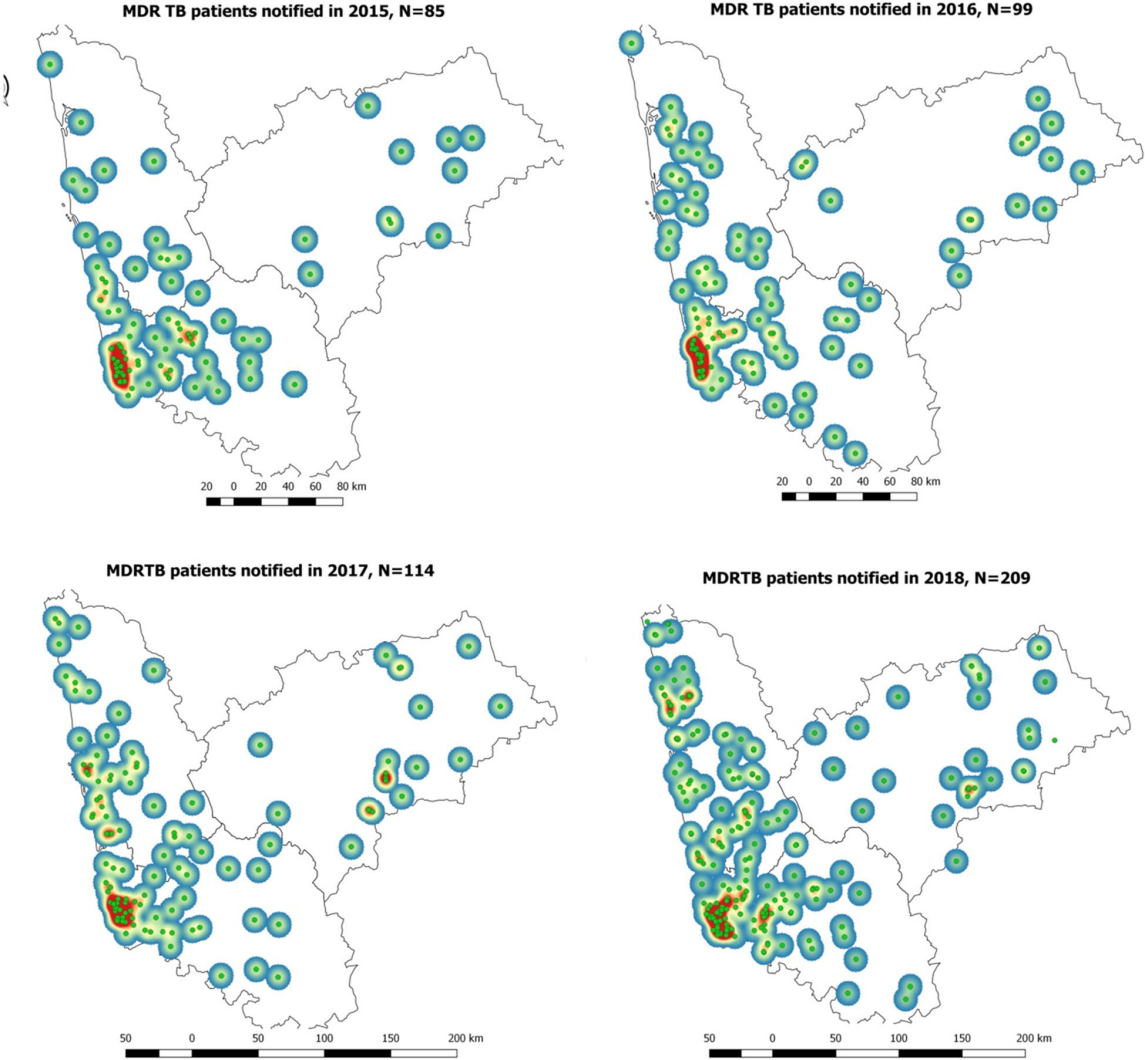
The heat map of drug-resistant TB patients notified during 2015 to 2018 in three selected districts of Karnataka, South India

**Fig. 4:**
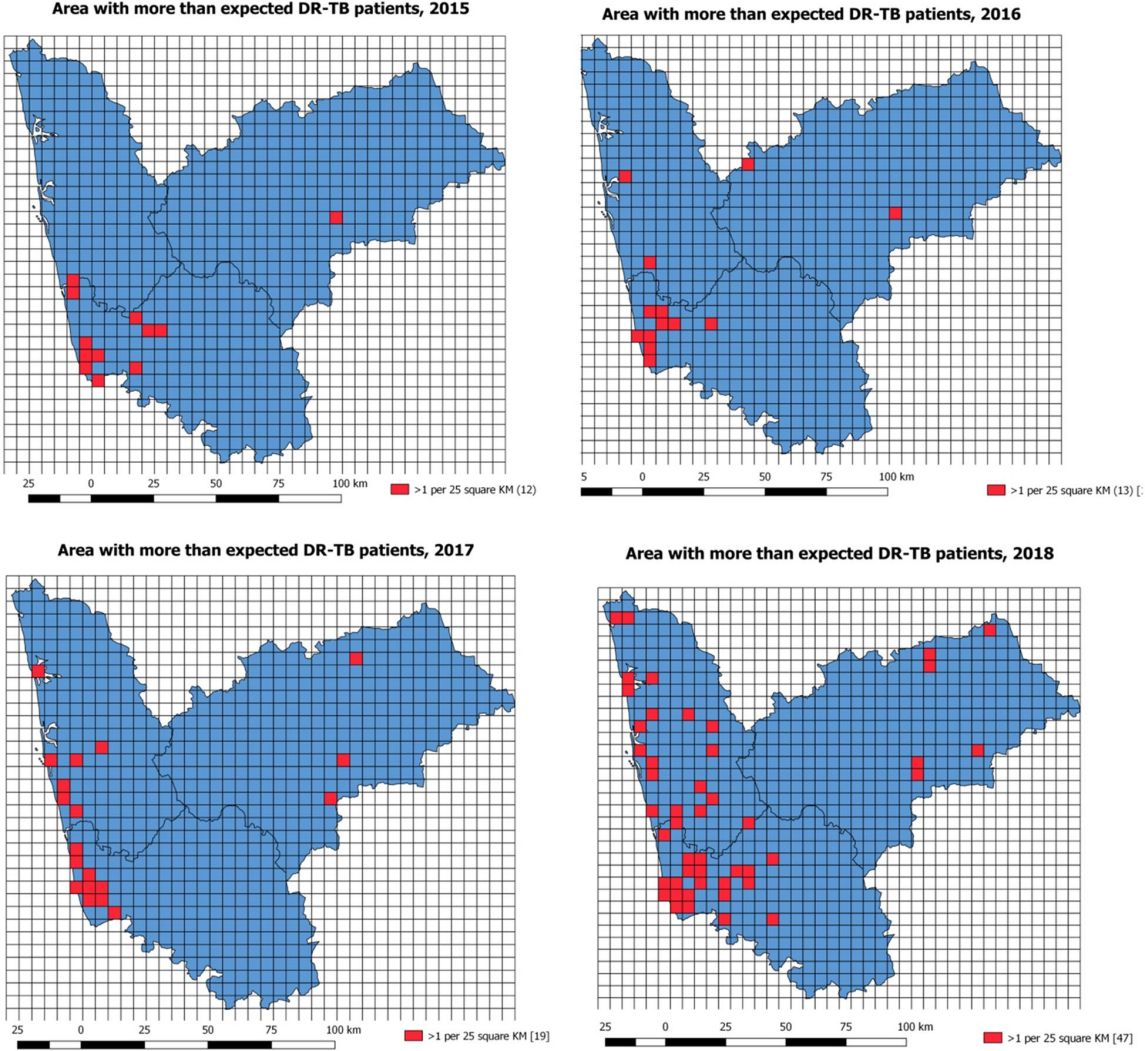
Map depicting the grids (25 km^2^) with more than the expected number of DR-TB patients

Figure 1 shows the spot map of DR-TB patients, and Fig. 3 depicts the year-wise “heat maps” generated for 2015 to 2018. The red color in the “heat map” indicates the area with the aggregation of DR-TB cases. The sum of the area of aggregation in square kilometers was 113.6, 95.2, 131.4, and 205.7 during 2015, 2016, 2017, and 2018, respectively. The number of rectangular grids of 25 km^2^ with more than the expected DR-TB patients (> 1) increased from 12 to 47 during the year 2015 to 2018 (Fig. 4).

**Fig. 5:**
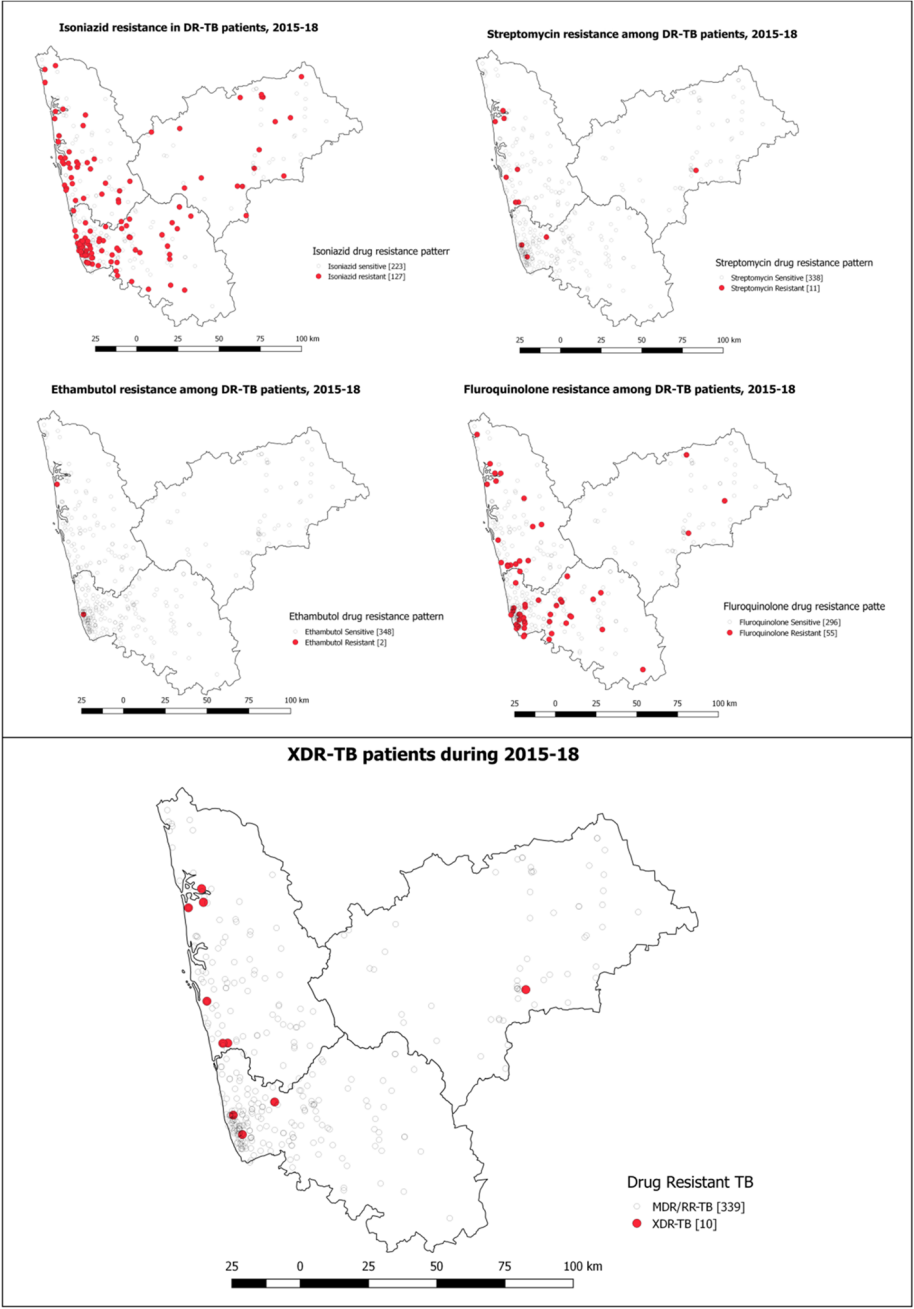
Spatial pattern of drug-resistant pattern of DR-TB patients notified during 2015 to 2018 in three selected districts of Karnataka, South India

Figure 5 and Table 2 show the geospatial and frequency distribution of resistance patterns of DR-TB patients, respectively. About 38% and 12% of the DR-TB patients had resistance to isoniazid and fluoroquinolones, respectively. The geospatial distribution did not depict the localized aggregation of similar resistance pattern. About 10 (2%) of the DR-TB patients had XDR-TB.

**Table 2:** Drug sensitivity pattern of DR-TB patients initiated on treatment under the national TB program in three selected districts of Karnataka during 2015 to 2018, *N* = 507

Drug sensitivity test	Frequency	Percentage
Resistance to isoniazid		
Yes	195	38.4
No	292	57.6
Not available	20	4.0
Resistance to ethambutol		
Yes	2	0.3
No	485	95.6
Not available	20	4.0
Resistance to streptomycin		
Yes	12	2.3
No	474	93.4
Not available	21	4.1
Resistance to fluoroquinolones		
Yes	59	11.6
No	429	84.6
Not available	19	3.7
XDR-TB		
Yes	10	1.9
No	476	93.8
Not evaluated	21	4.1

*Abbreviation*: *DR-TB* drug-resistant tuberculosis

## Discussion

This is the first study from India assessing the spatiotemporal trends of DR-TB patients using geocoded data of individual patients. The study showed spatial heterogeneity with an aggregation of DR-TB patients. Over the years (2015–2018), there was an increase in the number of DR-TB patients, the sum of the area of aggregation of patients, and the number of grid units with more than the expected DR-TB patients in the study districts. About one in ten DR-TB patients had resistance to fluoroquinolone.

There was an increase in the number of DR-TB patients in the study districts. The increasing trend was noted in each of the study districts. The annual TB reports have also reported an increase in notified DR-TB patients from 2015 to 2017. The increase might be either due to improved care delivery and adherence to protocols for the detection of DR-TB or due to the uninterrupted transmission of DR-TB. The massive increase in the number of DR-TB cases in 2018 could be due to introduction of the universal DST (DST made available for all TB patients). Improving the diagnostic services and making the services accessible might have increased the number of notified DR-TB cases. The other potential reasons for the increase in notification of DR-TB patients are imporved quality of DR-TB in public sector and also the disinterest of private providers in treating DR-TB patients within the private sector.

The previous studies in Peru [4] and Moldova [5] have reported spatial aggregation of DR-TB patients. Similar findings were seen in the current study. However, the study results are not comparable as the methods used in generating the spatial heterogeneity maps were different. The increase in the area of aggregation suggests there was a creation of local foci, which could be due to potential internal transmission. None of the previous studies explored the spatiotemporal aggregation.

The study has a few strengths. First, geocoding was done for all the notified cases during the study reference period as all the addresses could be extracted and traced back. Hence, there was no selection bias due to missing information in routine programmatic records. Second, the individual DR-TB patients were geocoded. This limited the aggregation bias of constructing heat maps using administrative boundaries and provided the opportunity to construct the heat maps based on the occurrence of an event. Third, the sample size was good for conducting spatial heterogeneity analysis and also spatiotemporal analysis. Fourth, double data entry and validation were used. This helped to limit the data entry errors and improved quality of data, more so with variables like latitude and longitude with five digits after the decimal point.

The study has a few limitations. First, the geocodes were approximated to village/ward or street level and thus failed to geotag the house of the patient. However, the villages in this part of the country have a small area [22] and therefore might not have led to gross imprecision in marking the DR-TB patients [22]. Second, the expected number of cases per 25 km^2^ was calculated based on the estimated TB incidence rate for India and averaging the population density in the three districts. This might have reduced the internal validity of the study findings. However, the incidence rate in the study districts might be lower than that of the country estimates, and thus, the expected number of DR-TB patients is an overestimate. The population across the district is not uniform, and there might be areas with a higher population density with a relatively higher number of expected DR-TB patients leading to underestimation. Thus, on the whole, the estimation of expected DR-TB patients per 25 km^2^ might be close to a real-world scenario. Third, there were no village- and ward-level shapefiles, which might have helped us to calculate the rates of occurrence of the DR-TB. Hence, we had to calculate the expected number of DR-TB patients, which might be inferior to rates. Fourth, the data series was available only for 4 years and had extreme variation. This restricted the use of time series analysis and prediction models. Fifth, we might have missed the undetected and not notified DR-TB patients. This could have limited the validity of heat maps as the not notified and undetected case might contribute to disease transmission but not accounted for in the analysis. Sixth, the study might have failed to capture the natural trend in the occurrence of the DR-TB as there was a change in the diagnostic algorithm, which could have influenced the case detection in the year 2018.

Despite several limitations, the study has a few implications. First, over the years, there was an increase in the number of cases and also in the area of aggregation of cases. The RNTCP needs to explore the potential reasons for this increase in DR-TB patients. If the programmatic change of upfront DST has brought this change, then this is a promising step in efforts towards control of DR-TB in India. However, if the increase is due to the transmission of the disease, then this is a cause for concern. Second, the geocoding and generation of heat maps provided insights on the aggregation of disease and the spread of disease to new areas over the years. The program can train the data entry operators to geocode the villages of DR-TB patients and generate maps to help in decision-making. Third, the utility of concentrated TB control efforts in areas with DR-TB aggregation on reducing the transmission and burden of DR-TB needs to be explored. Fourth, on moving towards the elimination of TB, there is a need for developing the geospatial monitoring indicators like “number of TB or DR-TB case per square kilometer” area and prioritize areas with high number of cases for intensified TB control activities.

## Conclusions

The increase in the number of DR-TB patients, area of aggregation, and grids with more than the expected count is a cause for concern. The NTP can use routine programmatic data to develop maps to generate local foci for targeted TB control activities.

## Data Availability

The datasets used and/or analyzed during the current study are available from the corresponding author on reasonable request.

## Abbreviations

CDST: Culture and drug susceptibility test
DMC: Designated microscopy center
DOTS: Directly observed treatment, short-course
DR-TB: Drug-resistant TB
DST: Drug susceptibility testing
HIV: Human immunodeficiency virus
LPA: Line probe assay
MDR-TB: Multidrug-resistant tuberculosis
MTB: Mycobacterium tuberculosis
NTP: National TB program
PHI: Peripheral health institute
PMDT: Programmatic Management of Drug-Resistant TB
Rif: Rifampicin
RNTCP: Revised National Tuberculosis Control Program
TB: Tuberculosis
TU: Tuberculosis unit
UDST: Universal drug susceptibility testing
WHO: World Health Organization
XRD-TB: Extensively drug-resistant tuberculosis
